# Insulin down-regulates cardioprotective SUR2A in the heart-derived H9c2 cells: A possible explanation for some adverse effects of insulin therapy

**DOI:** 10.1016/j.bbrep.2018.08.005

**Published:** 2018-09-06

**Authors:** Qingyou Du, Sofija Jovanović, Andriy Sukhodub, Yong Shi Ngoi, Aashray Lal, Marina Zheleva, Aleksandar Jovanović

**Affiliations:** a Division of Molecular and Clinical Medicine, Medical School, University of Dundee, United Kingdom; b University of Nicosia Medical School, Cyprus

**Keywords:** Insulin, Glucose, SUR2A, Heart

## Abstract

Some recent studies associated insulin therapy with negative cardiovascular events and shorter lifespan. SUR2A, a K_ATP_ channel subunit, regulate cardioprotection and cardiac ageing. Here, we have tested whether glucose and insulin regulate expression of SUR2A/K_ATP_ channel subunits and resistance to metabolic stress in heart H9c2 cells. Absence of glucose in culture media decreased SUR2A mRNA, while mRNAs of Kir6.2, Kir6.1, SUR1 and IES SUR2B were increased. 2-deoxyglucose (50 mM) decreased mRNAs of SUR2A, SUR2B and SUR1, did not affect IES SUR2A and IES SUR2B mRNAs and increased Kir6.2 mRNA. No glucose and 2-deoxyglucose (50 mM) decreased resistance to an inhibitor of oxidative phosphorylation, DNP (10 mM). 50 mM glucose did not alter K_ATP_ channel subunits nor cellular resistance to DNP (10 mM). Insulin (20 ng/ml) in both physiological and high glucose (50 mM) down-regulated SUR2A while upregulating Kir6.1 and Kir6.2 (in high glucose only). Insulin (20 ng/ml) in physiological and high glucose decreased cell survival in DNP (10 mM). As opposed to Kir6.2, infection with SUR2A resulted in titre-dependent cytoprotection. We conclude that insulin decreases resistance to metabolic stress in H9c2 cells by decreasing SUR2A expression. Lower cardiac SUR2A levels underlie increased myocardial susceptibility to metabolic stress and shorter lifespan.

## Highlights

• Insulin decreases cardioprotective SUR2A levels in embryonic heart H9c2 cells.
• Insulin decreases resistance to metabolic stress in H9c2 cells.
• Infection of H9c2 cells with SUR2A increases resistance of H9c2 cells to metabolic stress.
• Absence of glucose and 2-deoxyglucose had similar effects on H9c2 cells as insulin did.
• High glucose did not have any effect on SUR2A expression nor cellular resistance to stress.

## 1 Introduction

Diabetes mellitus is a group of metabolic diseases characterised by hyperglycemia resulting from defects in insulin secretion, insulin action, or both [1]. Insulin is a peptide hormone produced by pancreatic β-cells that regulates metabolism of carbohydrates and fats [2]. Insulin is the main therapy for type 1 diabetes (diabetes characterised by absolute insulin deficiency) and it is also sometimes used for therapy of type 2 diabetes (diabetes characterised by relative insulin deficiency and/or insulin resistance) [3]. In cardiac muscle, insulin promotes glucose uptake and its utilization via glycolysis and also participates in the regulation of long-chain fatty acid uptake and protein synthesis [4]. Traditionally, insulin has been considered to be cardioprotective [5], [6], [7], [8]. However, some more recent studies reported that insulin have cardiac effects that would not be expected from a cardioprotective hormone. In experimental animals, it has been demonstrated that insulin inhibits cardioprotection afforded by ischaemic preconditioning [9] while in patients with type 2 diabetes, concerns about negative cardiac events when insulin is used as a therapeutic have been raised [10]. A large meta-analysis suggested that insulin treatment is associated with a significantly higher short and long-term adverse cardiovascular outcomes after percutaneous coronary intervention compared to diabetic patients not treated by insulin therapy [11].

SUR2A belongs to a group of “atypical” ABC proteins as, although possessing a structure of an ABC protein, it does not seem to mediate transport [12]. In fact, SUR2A binds to inward rectifier Kir6.2 to form cardiac ATP-sensitive K^+^ (K_ATP_) channels. Increased level of SUR2A in the heart is demonstrated to 1) Protect myocardium against ischaemia-reperfusion [13], 2) Protect cardiomyocytes against hypoxia and other types of metabolic stresses [13], [14], [15], 3) Increase physical endurance [15], 4) Counteract ageing-induced increase in myocardial susceptibility towards hypoxia [16], 5) Counteract ageing-induced decrease in physical endurance (this effect could involve SUR2A effect on skeletal muscle as well, 9) and 6) Reprogram embryonic cardiomyocytes towards less differentiated stem cells [17]. Recently, we have uncovered that PI3K/Akt signalling pathway regulate SUR2A, ie. activation of PI3K/Akt up-regulates SUR2A and confers cardioprotection [23]. In addition to that, SUR2A expression seems to be regulated by intracellular ATP [24]. As insulin activates PI3K/Akt and regulates intracellular ATP by regulating metabolism of carbohydrates and fats [5], it is quite possible that this hormone could regulate SUR2A and, consequently, cardiac resistance to stress.

H9c2 cells are well-established experimental model that is similar to adult cardiomyocytes in crucial aspects of K_ATP_ channels structure, regulation and function; in both cell types express all seven K_ATP_ channel subunits [13], [14], [15], [16], [17], [18], [19], [20], [21], [22], [23], [24], [25] and increase in SUR2A increase numbers of fully functional K_ATP_ channels generating cellular phenotype more resistant to stress [13], [14]. Signalling pathway regulating K_ATP_ channel levels and mediating preconditioning and cardioprotection are similar between adult cardiomyocytes and H9c2 cells [23], [24], [25], [26], [27], [28], [29]. Therefore, we used this experimental model to examine whether glucose and insulin regulate SUR2A levels of cellular resistance to stress.

## 2 Methods

### 2.1 H9C2 cells and treatments with viral constructs

H9C2 cells Rat embryonic heart H9c2 cells (ECACC, Salisbury, UK) were cultured in a tissue flask (at 5% CO_2_) containing Dulbecco's modified Eagle's medium supplemented with 10% fetal calf serum and 2 mM glutamine and 1) 5 mM glucose added (control experimental group), 2) 20 ng/ml insulin and 5 mM glucose added (insulin experimental group), 3) 20 ng/ml insulin and 50 mM glucose added (insulin in high glucose experimental group). The cells were cultured in incubators (Galaxy, oxygen control model, RS Biotech, Irvine, UK) under those conditions for 24 h before experiments on them were performed. For some experiments H9C2 cells were infected with adenoviral constructs containing either green fluorescent protein (Ad-GFP), luciferase (Ad-luciferase), SUR2A (Ad-SUR2A) and Kir6.2 (Ad-Kir6.2). To infect H9C2 cells, a solution of recombinant adenovirus was mixed with culture medium, and cells were exposed to the virus with a multiplicity of 10 viral particles/cell (for Ad-GFP and Ad-luciferase), 0.2, 1 or 5 viral particles/cell (for Ad-Kir6.2) or 1, 3, 10, 30, 100 and 300 viral particles/cell (for Ad-SUR2A) for 48 h.

### 2.2 Real time RT-PCR

Total RNA was extracted from H9c2 cells using TRIZOL reagent (Invitrogen, Carlsbad, CA) according to manufacturer recommendations. Extracted RNA was further purified by RNeasy Plus Mini Kit (Qiagen, Crawley, UK) according to the manufacturer's instruction. Real time RT-PCR was performed as we described earlier [13], [14], [15], [16].

### 2.3 Cell survival assay

The survival of H9C2 cells were assayed using Multitox-Fluor Multiplex Cytotoxicity Assay (Promega). Briefly, H9C2 cells were plated in 96-well plate under conditions described in “H9C2 cells” section and after the incubation (24 h without viral constructs and 48 h with viral constructs) cells were washed out and DMEM containing 10% FCS and 10 mM 2–4 dinitrophenole (DNP) was added to each well. To measure cell survival 6 h later, the peptide substrate (GF-AFC) that can be cleaved only by live cells was added to the each well. Following 30 min-long incubation at 37 °C, plates were measured using 1420 Multibabel Counter (Victor) plate reader, with excitation at 370 nm and emissions of 480 nm. The percentage of live cells was calculated based on the intensity of fluorescence according to the manufacturer instructions.

### 2.4 Statistical analysis

Data are presented as mean±S.E.M, with n representing the number of independent experiments. Mean values were compared by the ANOVA followed by Student's *t*-test or by Student's *t*-test alone where appropriate using SigmaStat program (Jandel Scientific, Chicago, Illinois). P < 0.05 was considered statistically significant.

## 3 Results

### 3.1 Regulation of expression of K_ATP_ channel subunits by glucose and 2-deoxyglucose in H9c2 cells and cellular resistance to severe metabolic stress

**Fig. 1:**
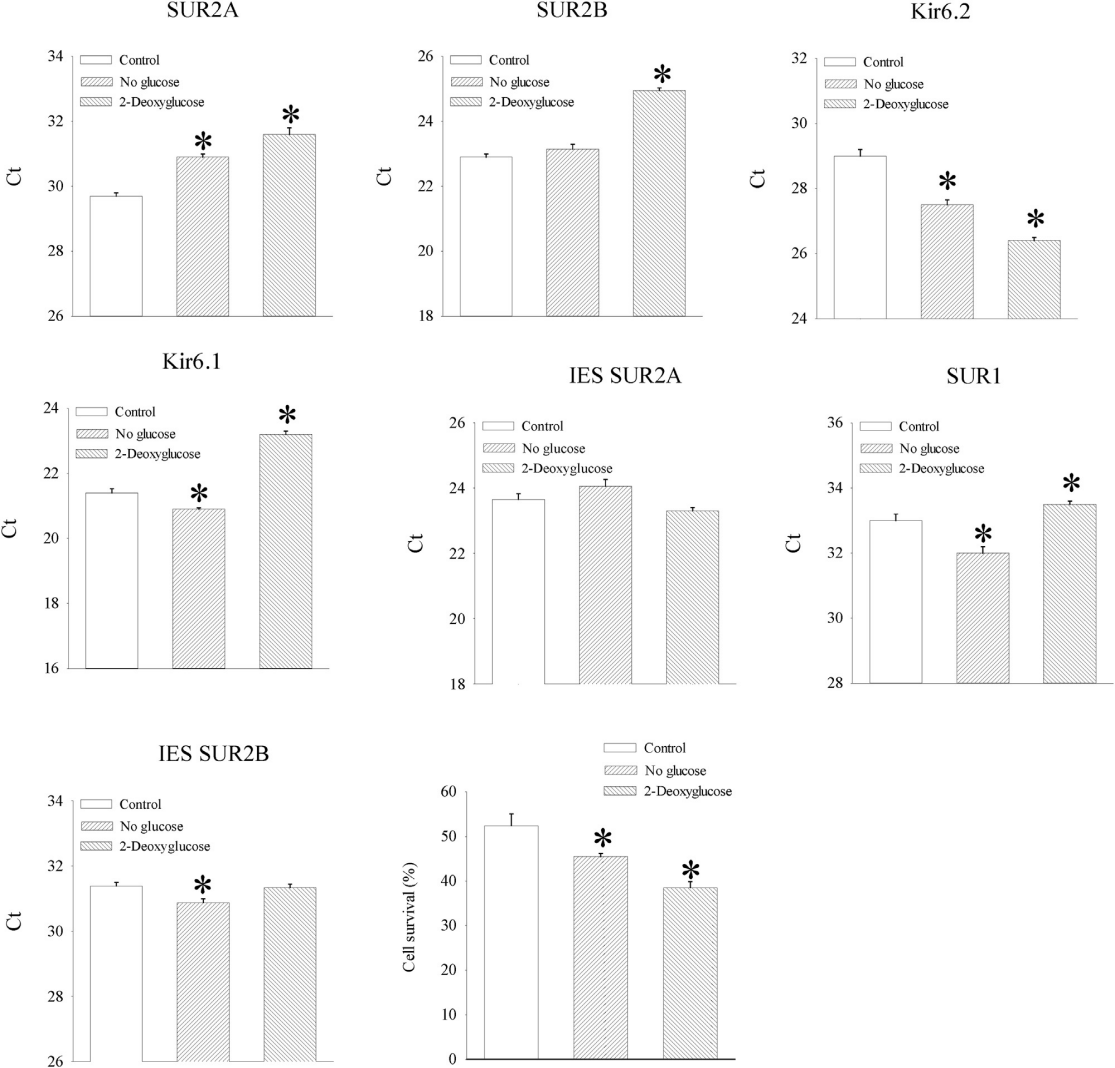
No glucose and 2-deoxyglucose down-regulate SUR2A in H9c2 cells and decrease cellular resistance to metabolic stress. Bar graphs represent cycling thresholds of the real time RT-PCR progress curves of K_ATP_ channel subunits as labelled and a bar graph (a graph on the right third row) showing a percentage of survival in control cells and cells cultured without glucose (no glucose) or cells cultured without glucose in the presence of 50 mM 2- deoxyglucose exposed to DNP (10 mM). Each bar represent mean±SEM (n = 6–7). *P < 0.05 when compared to control.

Glucose regulates the activity of sarcolemmal K_ATP_ channels via products of glycolysis that are direct ligands of these channels [30], [31]. Here, we have tested whether low glucose and inhibition of glycolysis regulate expression of SUR2A and other K_ATP_ channel subunits. Absence of glucose in culture media resulted in significant decrease of SUR2A mRNA levels (cycling threshold was 29.67 ± 0.11 for controls and 30.92 ± 0.11 for no glucose, P < 0.001, n = 6; Fig. 1) without affecting SUR2B and intra-exonic splicing (IES) SUR2A mRNA levels (SUR2B cycling threshold was 22.88 ± 0.06 for controls and 23.15 ± 0.14 for no glucose, P = 0.112, n = 6; IES SUR2A cycling threshold was 23.65 ± 0.17 for controls and 24.05 ± 0.21 for no glucose, P = 0.175, n = 6, Fig. 1), but increasing Kir6.2, Kir6.1, SUR1 and IES SUR2B mRNA levels (Kir6.2 cycling threshold was 28.98 ± 0.19 for controls and 27.50 ± 0.15 for no glucose, P < 0.01, n = 6; Kir6.1 cycling threshold was 21.43 ± 0.14 for controls and 20.90 ± 0.04 for no glucose, P < 0.01, n = 6; SUR1 cycling threshold was 32.98 ± 0.18 for controls and 32.03 ± 0.16 for no glucose, P < 0.01, n = 6; IES SUR2B cycling threshold was 31.38 ± 0.14 for controls and 30.87 ± 0.14 for no glucose, P = 0.018, n = 6, Fig. 1).

**Fig. 2:**
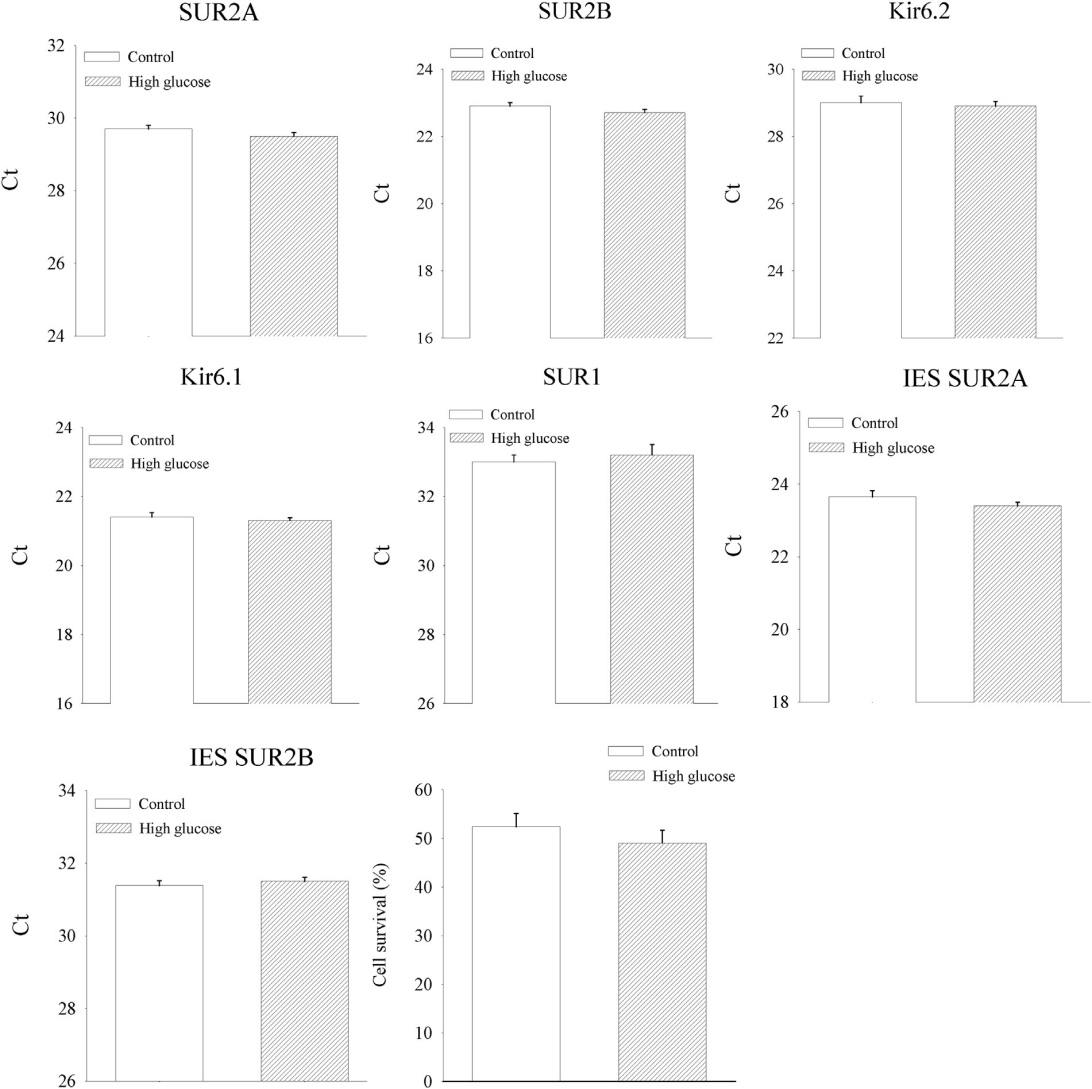
High glucose has no effect on SUR2A expression in H9c2 cells and cellular resistance to metabolic stress. Bar graphs represent cycling thresholds of the real time RT-PCR progress curves of K_ATP_ channel subunits as labelled and a bar graph (a graph on the right third row) showing a percentage of survival in cells cultured with 5 mM (control) or 50 mM (high glucose) glucose exposed to DNP (10 mM). Each bar represent mean±SEM (n = 6–10).

On the other hand, cells cultured in the presence of 2-deoxyglucose (50 mM) had decreased mRNA levels of SUR2A, SUR2B and SUR1 (SUR2A cycling threshold was 31.62 ± 0.20 for 2-deoxyglucose, n = 6, P < 0.01 when compared to controls; SUR2B cycling threshold was 24.95 ± 0.18 for 2-deoxyglucose, n = 6, P < 0.01 when compared to controls; SUR1 cycling threshold was 33.53 ± 0.11 for 2-deoxyglucose, n = 6, P = 0.029 when compared to controls; Fig. 1) without affecting levels of IES SUR2A and IES SUR2B (IES SUR2A cycling threshold was 23.32 ± 0.10 for 2-deoxyglucose, n = 6, P = 0.124 when compared to controls; IES SUR2B cycling threshold was 31.33 ± 0.11 for 2-deoxyglucose, n = 6, P = 0.790 when compared to controls; Fig. 1) and increasing levels of Kir6.2 (cycling threshold was 26.43 ± 0.09 for 2-deoxyglucose, n = 6, P < 0.01 when compared to controls; Fig. 1). Cell culturing in the absence of glucose significantly decreased cellular resistance to DNP (10 mM; cell survival was 52.4 ± 2.7% for controls and 45.5 ± 1.9%; P < 0.01; n = 7; Fig. 1). Exposure to 2-deoxyglucose (50 mM) significantly decreased cell survival in DNP (10 mM; cell survival was 38.5 ± 1.4% for 2-deoxyglucose; P < 0.01 when compared to controls and no glucose; n = 7; Fig. 1). Further, we have examined the effect of high glucose on K_ATP_ channel subunits and DNP-induced challenge. Exposure of cells to 50 mM glucose did not alter the levels of K_ATP_ channel subunits nor affected cellular resistance to DNP (10 mM; Fig. 2).

### 3.2 Regulation of expression of K_ATP_ channel subunits in H9c2 cells and cellular resistance to severe metabolic stress by insulin

**Fig. 3:**
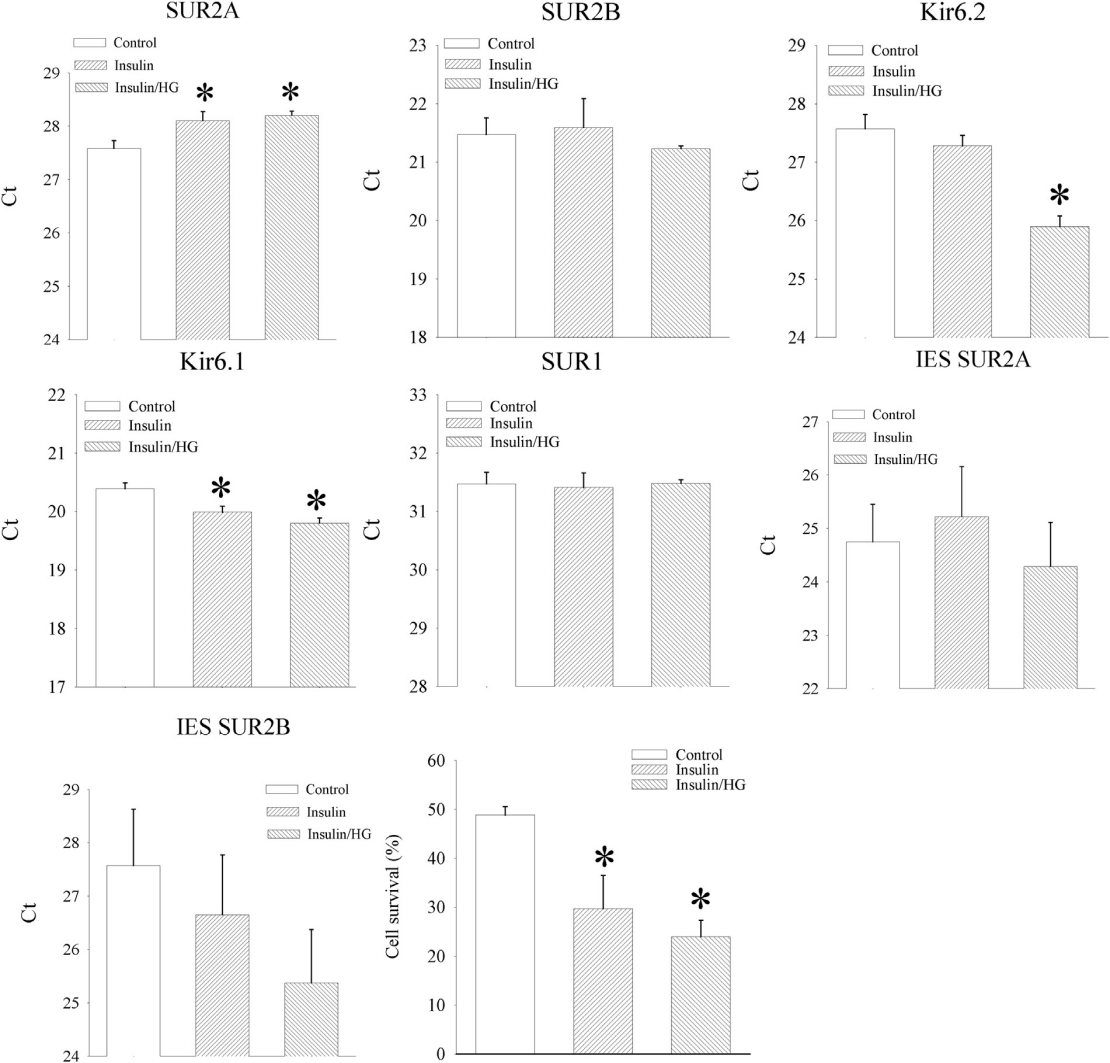
Insulin down-regulates SUR2A in H9c2 cells and decrease cellular resistance to metabolic stress. Bar graphs represent cycling thresholds of the real time RT-PCR progress curves of K_ATP_ channel subunits as labelled and a bar graph (a graph on the right third row) showing a percentage of survival in control cells and cells cultured with insulin (20 ng/ml) (insulin) or cells cultured with insulin (20 ng/ml) in the presence of 50 mM glucose exposed to DNP (10 mM). Each bar represent mean±SEM (n = 3–27). *P < 0.05 when compared to control.

Insulin activates PI3K/Akt signalling pathway which is known to up-regulate SUR2A [23]. When insulin (20 ng/ml) was applied in the presence of physiological glucose, it significantly down-regulated SUR2A (cycling threshold was 27.57 ± 0.15 without and 28.09 ± 0.17 with insulin, n = 12, P = 0.032; Fig. 3), did not affect expression of SUR2B, Kir6.2, SUR1, IES SUR2A and IES SUR2B (SUR2B: cycling threshold was 21.47 ± 0.29 without and 21.59 ± 0.48 with insulin, n = 10–12, P = 0.833; Kir6.2: cycling threshold was 27.57 ± 0.86 without and 27.28 ± 0.18 with insulin, n = 12, P = 0.175; SUR1: cycling threshold was 31.47 ± 0.20 without and 31.41 ± 0.25 with insulin, n = 12, P = 0.856; IES SUR2A: cycling threshold was 24.75 ± 0.65 without and 25.26 ± 0.94 with insulin, n = 8–11, P = 0.646; IES SUR2B: cycling threshold was 27.58 ± 1.06 without and 26.65 ± 1.12 with insulin, n = 8, P = 0.558; Fig. 3). A sole upregulated subunit was Kir6.1 (Kir6.1: cycling threshold was 20.39 ± 0.10 without and 19.99 ± 0.09 with insulin, n = 12, P < 0.01; Fig. 3). Application of insulin (20 ng/ml) in the presence of high glucose (50 mM) also down-regulated SUR2A (cycling threshold was 28.02 ± 0.08, n = 6, P < 0.01 when compared to control; Fig. 3) and the only difference between action of insulin in the presence of physiological (5 mM) and high (50 mM) glucose was in the effect on Kir6.2, where insulin in high glucose (50 mM) upregulated Kir6.2 (cycling threshold was 25.90 ± 0.18, n = 6, P < 0.01 when compared to those in 5 mM glucose; Fig. 3). Exposure of H9c2 cells to insulin (20 ng/ml) in either physiological (5 mM) or high (50 mM) glucose significantly decreased cell survival in DNP (10 mM; cell survival was 48.8 ± 1.7% and 43.5 ± 3.7% when cell were cultured without insulin in 5 mM and 50 mM glucose respectively, and 29.7 ± 6.7% and 24.0 ± 3.4% when cells were cultured in 20 ng/ml insulin in 5 mM and 50 mM glucose respectively, P < 0.01 when compared to corresponding experimental group without insulin, n = 3–27, Fig. 3).

### 3.3 Regulation of cell resistance to DNP by SUR2A and Kir6.2

**Fig. 4:**
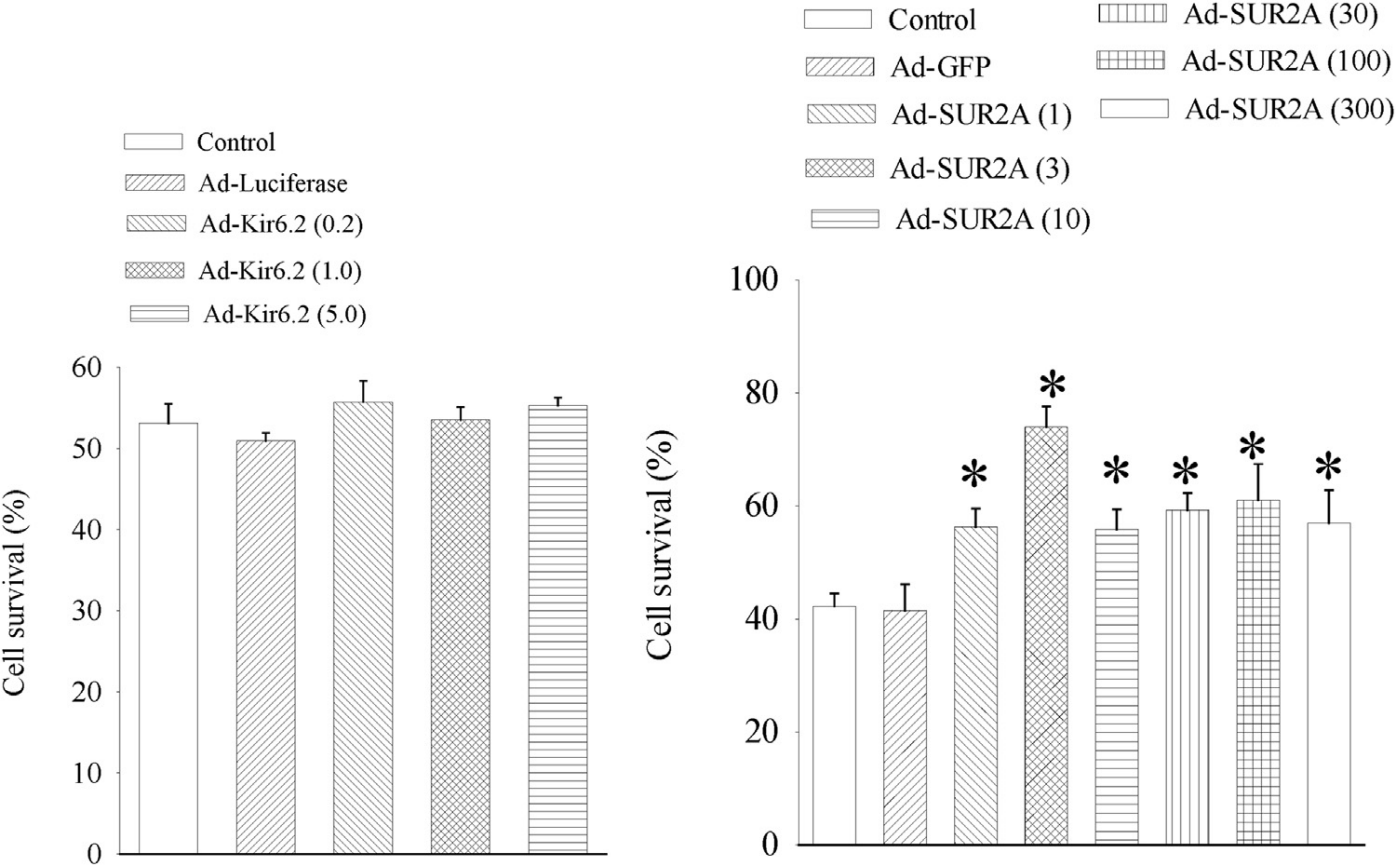
Infection with Ad-SUR2A, but not with Ad-Kir6.2, increases cellular resistance to metabolic stress. Bar graphs showing a percentage of survival in un-infected cells treated with 10 mM DNP (control) and 10 mM DNP-treated cells infected with SUR2A alone or Kir6.2 alone in different multiplicities of viral particles/cell as indicated on the graph symbols. Each bar represent mean±SEM (n = 5–9). *P < 0.05 when compared to control.

It has been shown previously that increased expression of SUR2A protects adult cardiomyocytes as well as H9c2 cells against different types of stresses [13], [14], [15], [16]. Here, we have tested whether infection with different titers of SUR2A alone or Kir6.2 alone would have any effect on survival of H9c2 cells when exposed to DNP (10 mM). Infection with Ad-Kir6.2 did not have any effect on cellular survival (Fig. 4), while infection with SUR2A demonstrated titre-dependent cytoprotection (Fig. 4).

## 4 Discussion

In the present study we have shown that insulin decreases resistance to metabolic stress in heart embryonic H9c2 cells by decreasing expression of SUR2A.

Insulin is known to activate PI3K/Akt signalling pathway and it was logically to expect that insulin would up-regulate SUR2A as this is what PI3K/Akt activation does [23]. However, the obtained effect was opposite, i.e. insulin down-regulated SUR2A. Insulin has been suggested to inhibit cardiomyocytes apoptosis [8], protects H9c2 cells against doxorubicin toxicity [32], and decrease the size of myocardial infarction in whole heart ischaemia-reperfusion model [33]. In contrast, it has been shown that insulin inhibits cardioprotection afforded by ischaemic preconditioning via Akt-dependent mechanisms [9]. In addition to that, there is a lot of clinical studies suggesting that insulin therapy had either no effect or worsened cardiovascular events [10], [11]. SUR2A regulates cellular resistance to metabolic stress in both adult cardiomyocytes and H9c2 cells [13], [14], [15], [16]. The underlying mechanism of cytoprotection afforded by SUR2A seems to be associated with SUR2A-mediated regulation of number of fully-assembled sarcolemmal K_ATP_ channels [13]. A cardiac phenotype with increased SUR2A and channel numbers is characterised by earlier activation of sarcolemmal K_ATP_ channels in response to metabolic stress [13], which is similar to those seen in preconditioning [34], [35]. Also, SUR2A regulates levels of subsarcolemmal ATP and this mechanism of cardioprotection is independent from the channel activity [14], [36], [37], [38]. Besides effects on cellular level, SUR2A also have major effects on whole organ and in vivo levels. Mice with non-targeted expression of SUR2A are characterised by increased physical endurance and slower decline in physical performance during ageing [15], [16] and they seem to live significantly longer than wild type mice [40]. So far, ageing is identified as the only condition that downregulates SUR2A and decreases cardiac resistance to stress [16], [41]. Observed insulin-induced decrease of SUR2A in H9c2 cells is in agreement with experimental/clinical studies reporting that insulin impairs cardioprotection. In further support of this notion is our finding that DNP, an inhibitor of oxidative phosphorylation, induced more cell deaths in insulin-treated cells than in untreated ones. This is consistent with previous findings that lower SUR2A levels are associated with increased susceptibility to ischaemia and β-adrenergic stress [15], [16], [39], [40], [41], [42]. Decrease in myocardial SUR2A could explain negative cardiac events observed in insulin-treated diabetes type 2 patients [10], [11] and shorter lifespan [43].

It is well established that insulin regulates expression of many genes [2], [3], [4], [5]. Here, we have shown that it also up-regulates Kir6.2. What are consequences of such an effect is yet unknown, but it is certain that, as opposed to SUR2A, an increase of Kir6.2 alone does not regulate cellular resistance to stress. Studies so far demonstrated that cellular resistance follow up SUR2A levels even when expression of other genes is altered [13], [14], [15], [16], [42]. Apart from SUR2A and Kir6.1, insulin did not affect expression of any other gene encoding sarcolemmal or mitochondrial K_ATP_ channel-forming subunit showing that those are not insulin targets.

Many studies in the past demonstrated that the effect of insulin is influenced by the presence of glucose [2], [3], [4], [5]. Therefore, we have assessed whether high glucose would affect insulin action on K_ATP_ channel subunits expression. It has been previously shown that high glucose activates K_ATP_ channels in cardiomyocytes via products of glycolysis that act as channel openers [30], [31]. However, high glucose did not affect expression of any of K_ATP_ channel-forming subunits nor had any major influence on insulin-mediated regulation of channel subunits or cell survival. These finding suggest that the observed insulin effect was glucose-independent. In contrast, a lack of any effect on K_ATP_ channel subunits expression by high glucose, no glucose or inhibition of glycolysis down-regulated SUR2A. These findings were not that surprising when considering that intracellular ATP regulates SUR2A expression [24]. As inhibition of glycolysis decrease intracellular ATP, our findings that it also decreases SUR2A levels perfectly fits into previously published findings that SUR2A levels follow intracellular ATP levels [24]. However, it is surprising that insulin would have similar effect on SUR2A expression and cellular susceptibility to metabolic stress as no glucose and inhibition of glycolysis have. It is also interesting to note that insulin regulated levels of SUR2A alone without affecting levels of SUR2B, IES SUR2A and IES SUR2B. As all these proteins are products of a single gene, this would imply that insulin could act on splicing mechanism as well. However, this is not certain. It is interesting to note that many conditions regulate SUR2A or SUR2B or IES SUR2A/B levels alone without affecting other products of ABCC9 gene [15], [16], [22], [39], [44]. Thus, a selective regulation of a single ABBC9 product is not unusual at all and this is a phenomenon worthwhile to be understood.

Insulin-induced decrease in cardiac SUR2A is previously unrecognised effect that should be seriously taken into account in the future. Lower SUR2A levels are likely to be associated with increased myocardial susceptibility to metabolic stress, decreased physical endurance and lifespan [15], [39]. Recognising decrease in myocardial SUR2A as a consequence of therapy with insulin would be crucial, as there are safe compounds/strategies that could be clinically implemented to up-regulate SUR2A [15], [39].
